# The 2011 Tohoku-oki tsunami-induced sediment remobilization on the Sendai shelf, Japan, from a comparison of pre- and post-tsunami surface sediments

**DOI:** 10.1038/s41598-021-87152-8

**Published:** 2021-04-12

**Authors:** Ken Ikehara, Tomohisa Irino, Yoshiki Saito

**Affiliations:** 1 Geological Survey of Japan, National Institute of Advanced Industrial Science and Technology (AIST), Tsukuba Central 7, 1-1-1 Higashi, Tsukuba, 305-8567 Japan; 2 Faculty of Environmental Earth Science, Hokkaido University, Kita-10 Nishi-5, Kita-ku, Sapporo, 060-0810 Japan; 3 Estuary Research Center, Shimane University, Nishikawatsu-cho 1060, Matsue, 690-8504 Japan

**Keywords:** Natural hazards, Environmental impact

## Abstract

Tsunamis are generally considered to disturb the seafloor, rework surface sediments, and change seafloor environments. However, the response of the seafloor to such extreme wave events has not been fully elucidated. Herein, we compare the surface sediments before and after the 2011 Tohoku-oki tsunami on the Sendai shelf and demonstrate that both sandy and muddy sediments were significantly reworked on the shelf. Muddy sediments (> 10 cm thick) were redeposited as graded mud with no or little bioturbation, characterizing the offshore muddy tsunami deposit, while well-sorted sand was found as the sandy tsunami deposit. This redeposited layer could also be retained in the shelf mud sequence. The results imply that the high friction velocity of the tsunami wave and its long-term effect on Sendai Bay might contribute to the large sediment reworking. Part of the resuspended mud moved offshore to the slope area as turbidity currents. Thus, the tsunami is an important mechanism not only for shelf sedimentation but also for deep-sea sedimentation along active plate margins. The detection of ^134^Cs derived from the Fukushima Daiichi Nuclear Power Plant accident in the redeposited mud indicates that the suspended shelf water state was maintained for some days after the tsunami.

## Introduction

The 2011 off the Pacific coast of Tohoku (Tohoku-oki) earthquake (Mw > 9: epicenter location 38.10° N, 142.85° E; hypocenter depth 24 km) occurred on March 11, 2011^1^ and caused a catastrophic tsunami (Tohoku-oki tsunami) with a run-up height in northern Honshu over 40 m^2^. This extensive run-up of the tsunami created onshore tsunami deposits along the Pacific coast of the Japanese islands^3–8^. As the shear velocity of the tsunami wave increases with decreasing water depth^9,10^, the tsunami can transfer marine surface sediments on the shelf to the upper slope, leading to the formation of deposits onshore and/or in marine environments. This phenomenon could be supported by earlier findings, as sublittoral to bathyal microfossils have been detected in some onshore and shallow bay tsunami deposits created from previous tsunamis^11,12^. However, no marine evidence was found in the onshore tsunami deposits created by the 2011 Tohoku-oki tsunami in the Sendai Plain^4^. Prior to the 2011 Tohoku-oki tsunami, the reports on the effect of tsunami on the marine surface sediments were very limited^13–16^. In contrast, several studies have reported the occurrence of coastal and offshore sediment remobilization due to the 2011 Tohoku-oki tsunami in large areas along the Pacific coast of Tohoku^17–22^. Nevertheless, the response of marine surface sediments to tsunami has not yet been fully understood, as it varies depending on the local environmental conditions and parameters such as bathymetry, coastline morphology and environment, grain size and composition of the surface sediment, size and direction of the tsunami wave. Therefore, to better understand the offshore tsunami deposits and their formation processes and to quantify the impact of tsunami on the seafloor, comparative studies on surface sediments before and after tsunamis in as many locations as possible are necessary. Furthermore, some recent studies have indicated the possibility of tsunami-induced sediment transport from shelves to slopes^17,18,21,22^. Thus, it is important to consider the influence of shelf sediment remobilization by tsunamis on deep-sea sedimentation.

The Geological Survey of Japan, AIST conducted a surface sediment sampling campaign on the Sendai Bay shelf in 1985^23–25^. Based on the sufficient preliminary data, we decided to collect surface sediment samples from the same sites of the 1985 survey to understand the changes in the surface sediment caused by the 2011 Tohoku-oki tsunami.

### Sendai Bay: bathymetry, pre-tsunami surface sediments, and the 2011 tsunami

**Figure 1:**
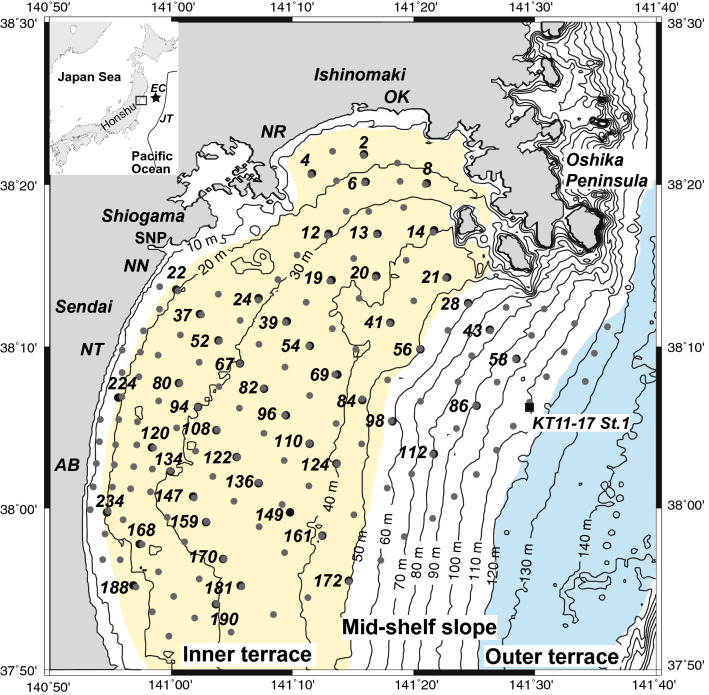
Location and bathymetry of Sendai Bay and surface sediment sampling locations. Black solid circle; the 2012 sediment sampling location, gray solid circle; the 1985 sediment sampling location. Yellow; inner terrace, blue; outer terrace. AB; Abukuma River mouth, NT; Natori River mouth, NN; Nanakita River mouth, NR; Naruse River mouth; OK; Old Kitakami River mouth; SNP; Sendai New Port, JT; Japan Trench; EC; Epicenter of the 2011 Tohoku-oki earthquake.

Sendai Bay is an open bay facing the Pacific Ocean (Fig. 1) comprising two strand plains to the west (Sendai Plain) and north (Ishinomaki Plain) and a rocky coast between the two plains. The shelf of Sendai Bay is 80 km wide and is composed of two terraces and a mid-shelf slope. The shallow inner terrace has water depths of 15–50 m, while a distinct and steep shoreface slope exists along the landward margin of the inner terrace along the two strand plains. The shoreface is composed of well-sorted sandy sediments with low mud content that reflect the high wave actions^24,25^. Silt is distributed in the northern (northern inner shelf mud; NISM) and northwestern (northwestern inner shelf mud; NWISM) parts of the inner terrace and along the mid-shelf slope (mid-shelf mud; MSM), while moderate to poorly sorted and sometimes gravelly sand (central gravelly sand; CGS) is distributed in the central to southern part of the inner terrace. In addition, some parallel and cross laminated sand layers with upward fining grading structures were intercalated in the muddy sequences collected from the NISM and NWISM areas, and considered as the storm sand layers^24,25^.

The outer terrace has a water depth of 120–145 m, while a small mid-shelf slope with a maximum gradient of 0.3°, which consists of several small canyons, can be found between the inner and outer terrace. Although various paleo-channels have been recognized below the present seafloor around the canyons^25^, the relationship between the channels and canyons is still unclear^26^. The outer terrace is covered with muddy sand and/or sandy mud, while relict shore-connected sand ridges can be found on the outer terrace^24,25^. The shelf break occurs at a water depth of ~ 145 m.

Wave gauge data indicated that the water level at Sendai New Port (SNP in Fig. 1) initially gradually decreased after the 2011 Tohoku-oki earthquake and then increased ~ 50 min after the earthquake. Although no instrumental record of the maximum height is available due to damage of the wave gauge by the 2011 tsunami, wave inversion has suggested that the maximum water level reached ~ 8 m at ~ 70 min after the earthquake^27^. The following tsunami waves were inferred to be 3–5 m high. The maximum inundation height was 19.5 m, and the mean inundation height near the shoreline was ~ 10 m^28^. Numerical simulation has indicated that the calculated shear velocity of the tsunami at the Sendai Bay mouth at the water depths of 90–130 m was 0.03–0.04 m s^−1^, which can move sand grains^9^. Another simulation suggested that the tsunami shear force was large enough to move sand grains in Sendai Bay in water depths less than 150 m, except for gravelly bottoms^10^.

## Results

### Post-tsunami surface sediment distribution

**Figure 2:**
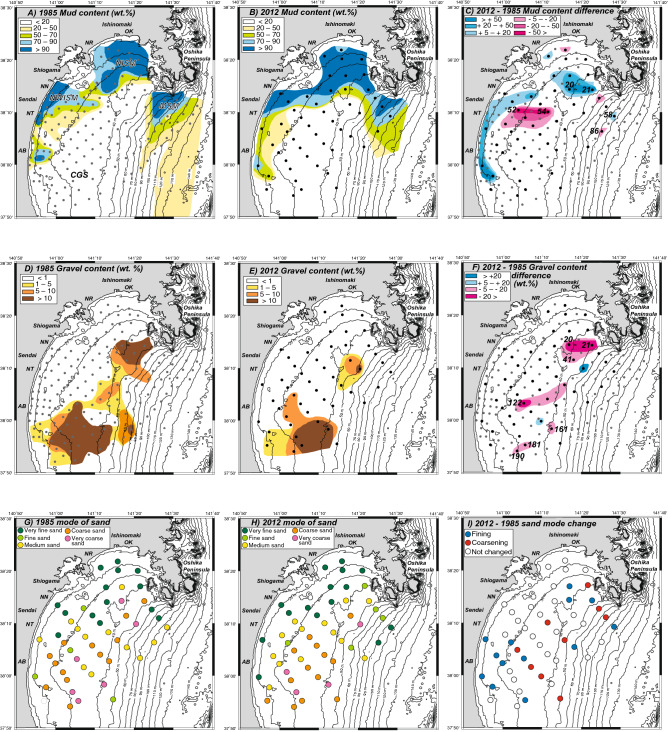
Spatial distribution of mud content (wt.%), gravel content (wt.%) and mode of sand fraction on the pre- (in 1985; **A**,**D**,**G**) and post-tsunami (in 2012; **B**,**E**,**H**) Sendai shelf, and these differences (**C**,**F**,**I**). *NISM* northern inner shelf mud, *NWISM* northwestern inner shelf mud, *MSM* mid-shelf mud, *CGS* central gravelly sand.

Approximately one-third of the sampling sites from the 1985 survey^23–25^ were resampled from August to September 2012, namely ~ 1.5 years after the 2011 Tohoku-oki earthquake and tsunami. Fifty surface sediment samples were collected from the inner to middle shelf of Sendai Bay (Fig. 1 and Supplementary Table S1). By comparing the spatial distribution of the mud content between the pre- and post-tsunami samples, it was indicated that the surface sediment distribution is slightly changed after the Tohoku-oki tsunami (Fig. 2 and Supplementary Table S2). Specifically, the distribution of the shelf mud (NISM and NWISM) expanded to west of Oshika Peninsula, and while CGS decreased slightly to south and west (Fig. 2C). The mud content increased > 50 wt% at Sites 20 and 21, while the mud content of MSM increased at Site 58 but decreased at Site 86. Another difference was observed near the mouths of the Abukuma and Natori rivers (AB and NT in Fig. 1, respectively), where muddy sediments were detected in the post-tsunami survey (Fig. 2C). In contrast, the mud contents decreased (> 50 wt%) east of the Natori River mouth (Sites 52 and 54; Fig. 2C). Although the CGS mud contents did not change, the gravel content and mode of sand fraction was different at some sites (Fig. 2D–I; Supplementary Table S2). Particularly, the gravel content decreased slightly but widely in the CGS (Fig. 2F), while a significant reduction was observed at Sites 20, 21 and 41 west of the Oshika Peninsula (Fig. 2F), where the mud contents also changed significantly. A reduction in the gravel content was observed at the CGS Sites 122, 161, 181, and 190 (Fig. 2F). The mode of sand fraction changed mainly in the peripheral part of the CGS area, especially east of the Abukuma River mouth and west of the Oshika Peninsula (Fig. 2I). Although fining changes occurred near the Abukuma River mouth (Fig. 2I), no systematic changes of the sand mode or fining and coarsening changes were identified in the nearby sites.

### Subcore lithology and sedimentary structures

**Figure 3:**
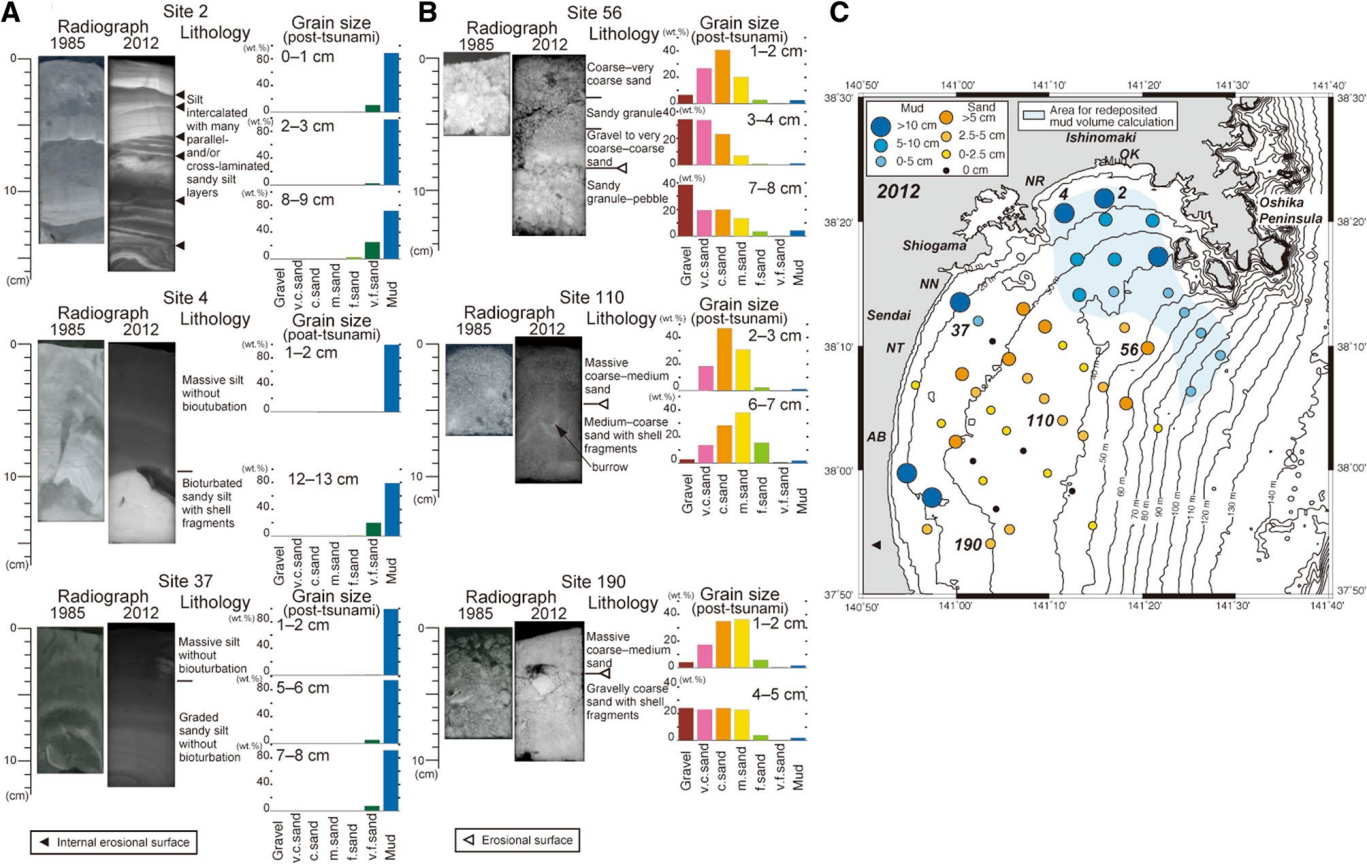
Comparison of sedimentary structures of the 1985 and 2012 sample, and description and grain-size distribution of 2012 subcore sample (**A**,**B**), and thickness of the upper homogeneous mud and sand (**C**). Sites 2 and 4 from the NISM, and 37 from the NWISM, Sites 56, 110 and 190 from the CGS.

Changes in the sedimentary structures of the surface sediments were widely recognized (Fig. 3). The most obvious change was observed in the NISM and NWISM. Specifically, a homogeneous structure with no or little evidence of bioturbation except for one or a few burrows from the core top (seafloor) was a significant characteristic of the post-tsunami (2012) cores of NISM and NWISM (Fig. 3A), whereas strong bioturbation was mainly observed in the pre-tsunami (1985) cores of NISM and NWISM (Fig. 3A), indicating that the sedimentary structure of NISM and NWISM was completely changed. Furthermore, some post-tsunami cores showed fining upward structures and intercalation of the coarse-grained layers (Fig. 3A). For example, the X-ray radiodensity at Sites 4 and 37 decreased (darker in the images), suggesting a fining upward grading trend (Fig. 3A). In addition, the coarse silt at the bottom of the Site 37 core had parallel laminations, while multiple intercalations of the coarse-grained layers with erosional bases were observed at Site 2 (Fig. 3A). Moreover, the homogeneous or graded mud without bioturbation was thick near the coast (> 17 cm at Site 2, > 14.5 cm at Site 14, 14 cm at Site 22, and 11 cm at Site 4) and became thinner in the offshore direction (Fig. 3C).

**Figure 4:**
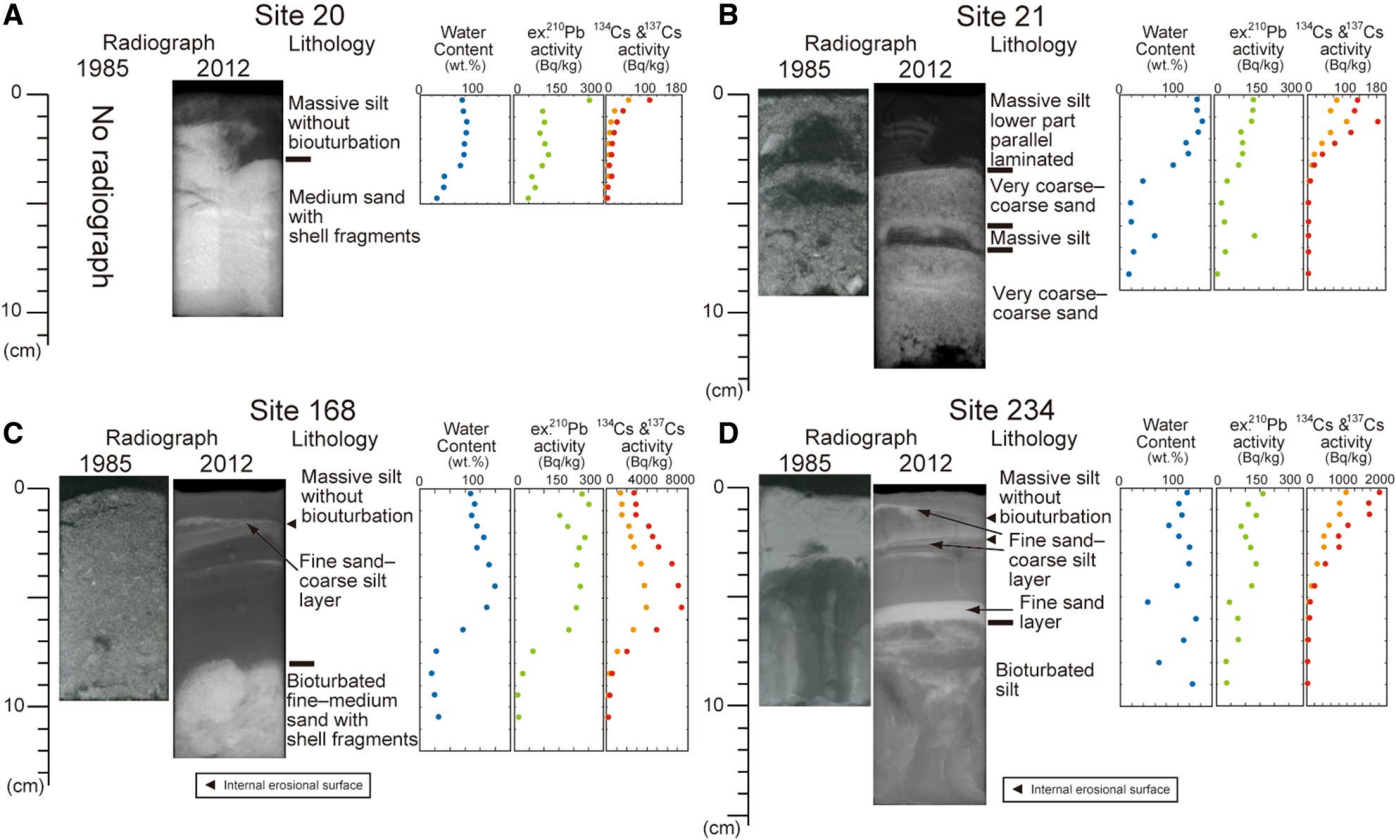
Comparison of sedimentary structures of the 1985 and 2012 sample, and description of 2012 subcore sample, and the profiles of water content (blue), excess ^210^Pb (green), ^134^Cs (orange), ^137^Cs (red) activities at Sites 20 (**A**), 21 (**B**), west of the Oshika Peninsula, and at Sites 168 (**C**) and 234 (**D**), off Abukuma River mouth.

Another large change in the sediment lithology was detected near the Abukuma River mouth. Well-sorted fine sand with low mud content was found in 1985 at Site 168 (Fig. 4C), whereas in 2012, coarsening upward mud with cross laminations covered the very fine sand (Fig. 4C) and an internal erosional surface was observed in the mud. Similar coarsening upward mud with cross laminations and internal erosional surface was also collected from Site 234 (Fig. 4D) and a thin fine sand layer was observed at the base of the mud. Beneath the fine sand layer, a vertically arranged mud was also found, similar to the mid-lower part of the pre-tsunami core (Fig. 4D). However, very fine sand covered the vertically arranged mud in 1985. Therefore, we concluded that the deposition of 5–10 cm thick mud occurred after 1985.

Most of the gravelly sand in CGS had the same sedimentary structures. However, at Sites 20 and 21 near the boundary between the NISM and NWISM and the CGS, the sand was covered by silt (Fig. 4A,B). Moreover, the lamination found in the newly deposited mud in the core of Site 21 suggested the effect of the flow during deposition. Furthermore, the intercalation of similar thin mud layers in the sand at Site 21 was found in both the pre- and post-tsunami cores (Fig. 4B).

Most of the cores from CGS also showed a change in the sediment lithology and sedimentary structures (Fig. 3B). Massive coarse–medium sand was occasionally found at the uppermost part of the subcores. Sometimes, burrows were observed in the lower sandy sediments, but no burrow was found in the upper sandy sediments. The boundary between the upper and lower sandy sediments was sharp, while erosional surface cut the burrow structures of the lower sandy sediments (Fig. 3B). The upper sandy sediments were usually better sorted than the lower ones (Fig. 3B). The thickness of the upper sandy sediments was only 1– ~ 8 cm (Fig. 3C). Well-sorted medium sand was found at Site 122, although in the 1985 survey, poorly sorted gravelly sand was identified at this site (Supplementary Fig. S1A). Well-sorted coarse–very coarse sand with homogeneous structure covered the poorly sorted gravelly sand at Site 56 (Fig. 3B). A similar gravelly sand was also found at the seafloor in 1985 (Fig. 3B). At Site 190, homogeneous coarse–medium sand covered the gravelly coarse sand (Fig. 3B), where the gravel content of the surface sediments was reduced. At Site 80, bioturbated very fine sand changed to parallel laminated very fine sand with some small burrows near the core top (Supplementary Fig. S1B), implying that at least a part of the sand was reworked on the Sendai inner shelf. Moreover, the relatively well-sorted grain size of the sediment suggested that the sediment grains were also sorted during transportation and deposition.

### Radiocesium and excess ^210^Pb activities

The high activities of excess ^210^Pb (58–250 Bq kg^−1^ for Site 20 and 40–128 Bq kg^−1^ for Site 21; Fig. 4A, B and Supplementary Table S3) suggest that the newly deposited mud originated from the near surface (recent) sediments. The lack of bioturbation and presence of ^134^Cs (4–53 Bq kg^−1^ for Site 20 and 2–85 Bq kg^−1^ for Site 21) and ^137^Cs (75–104 Bq kg^−1^ for Site 20 and 4–161 Bq kg^−1^ for Site 21) in the covering mud (Fig. 4A,B and Supplementary Table S3) indicate that the covering mud was young and deposited after the Fukushima Daiichi Nuclear Power Plant (FDNPP) accident, as the release of ^134^Cs into the atmosphere from the FDNPP started on March 12, 2011, peaked on March 15 and continued to March 23, 2011^29^.

Moreover, the radioactivity measurements revealed very high activities of ^134^Cs (92–3864 Bq kg^−1^ for Site 168 and 3–967 Bq kg^−1^ for Site 234) and ^137^Cs (172–7336 Bq kg^−1^ for Site 168 and 8–1815 Bq kg^−1^ for Site 234; Fig. 4C,D and Supplementary Table S3) and relatively high activity of excess ^210^Pb (7–247 Bq kg^−1^ for Site 168 and 20–156 Bq kg^−1^ for Site 234; Fig. 4C,D and Supplementary Table S3). The activities of ^134^Cs and ^137^Cs were > 20 times higher than those of the redeposited mud of the NISM at Sites 20 and 21. In addition, the ^134^Cs/^137^Cs ratios in all samples ranged from 0.42 to 0.62, which were lower than the theoretical ratio (0.63) on September 1, 2012, considering an initial ratio of 1 on March 15, 2011^30^, and then, the reported ratios of the surface sediments around Fukushima^31^.

## Discussion

In addition to the grain size (mud content and sand fraction mode) of the surface sediments, the sedimentary structures on the Sendai inner shelf were also significantly changed between 1985 and 2012. However, such changes in sedimentary structures were not observed in the 1 year-interval resampling comparison of NISM and NWISM^24^. The graded mud in NISM and NWISM suggests that the mud was most likely deposited by suspended water, such as the upper portion of the fine-grained turbidite^32^. Similar muds have been reported in distal cores from coastal lakes inundated by large tsunamis^33,34^. However, the lack of the basal sandy part did not allow the estimation of the current conditions and/or the degree of lateral transport, whereas the parallel laminations in some basal coarse-grained layers (Fig. 3A) indicate the effect of flow during deposition. In contrast, no bioturbation was observed, suggesting that the graded mud in the sediments was very recent without sufficient time for the macrobenthos to burrow into and mix them. Although several large storm and earthquake events occurred between 1985 and 2012 (Supplementary Tables S4 and S5), the detection of ^134^Cs also suggests that the redeposited mud (Sites 20 and 21; Fig. 4A,B) was formed after the FDNPP accident, while the lack of bioturbation in the redeposited mud indicates that the benthos activity (burrowing) did not lead to the subsurface occurrence of ^134^Cs. Although large ground shaking by the 2011 earthquake might have contributed to surface sediment resuspension^22^, the following tsunami-induced shear velocity resulted in large-scale erosion and the resuspension of shelf mud. Since the release of radionuclides to the atmosphere from the FDNPP started on March 12, 2011 and peaked on March 15, 1 and 4 days after the earthquake and tsunami, respectively, the particles must have been suspended in the shelf water for at least several days. Considering the occurrence of turbid bottom water a few weeks to months after earthquake^35,36^, the suspended shelf water condition might have been maintained for a few weeks or more. Therefore, the graded mud was most likely formed after the 2011 tsunami.

Radioactive Cs found in high concentrations in the fine-grained portion of the surface sediments (Fig. 4). The same trend has been reported in the 2011 event deposits on the outer shelf of Sendai Bay^18,22^. As Cs can be strongly absorbed on mica-like clay minerals^37–39^, radioactive Cs might also absorb on the fine-grained clay minerals in the suspended shelf water. In addition, due to the higher concentration of total organic carbon in NISM and NWISM than that in CGS^24,40,41^, highly radioactive Cs might also absorb on the organic matters^42^ in the suspended materials. The thickness of the graded mud without bioturbation ranges from a few cm to more than 17 cm (Fig. 3C). As the stable carbon isotopes and C/N ratios of the surface sediments before and after 2011 were similar^40,41^, the muddy sediments of NISM and NWISM were probably resuspended and redeposited on the Sendai inner shelf. The ^134^Cs/^137^Cs ratios (Supplementary Table S3) also suggest the remobilization of the young surface sediment. Using a half-life of ^134^Cs (2.0 years) and ^137^Cs (30.1 years), original ^134^Cs/^137^Cs ratios on April 1, 2011, when radiocesium was released from the FDNPP, were calculated as ~ 0.8 (0.67–0.98). However, since the initial ^134^Cs/^137^Cs ratio of the FDNPP derived radiocesium was ~ 1^30^, the additional ^137^Cs amount in the observed ratios should be explained. The young surface sediments in NISM, NWISM and MSM were probably resuspended and provided the extra ^137^Cs in the event mud. The high and relatively constant activities of excess ^210^Pb in the event mud (Fig. 4) also suggest the resuspension of the young surface sediment. In addition, the new mud covering west of the Oshika Peninsula at Sites 20 and 21 indicated that the resuspended muddy particles were deposited across the boundary between the previous NISM/NWISM and CGS. The volume of resuspended mud in NISM and MSM was calculated as > 20 × 10^6^ m^3^ from the redeposited mud thickness distribution (Fig. 3C). However, the volume may be larger than estimated, because part of the resuspended mud was moved offshore to the outer shelf and further to the slope area following the turbidity currents^17,18,21,22^.

The sediment remobilization by the 2011 tsunami was also recognized in CGS based on the changes in the gravel contents and mode of sand fraction. The new deposition of massive sand over a sharp surface was observed at some sites (Fig. 3B and Supplementary Figs. S1E and F), while significant changes occurred along the 30 m water depth contour (Fig. 3C). The massive structure without burrows in the newly formed surface sand layers suggests that the layers are recent deposits and that there has been insufficient time for the macrobenthos to burrow into them. This area was also consistent with the area of high total energy of shear force [> 0.5 (N m^−2^)(m s^−1^)(s)] by the 2011 tsunami, thus reflecting the high wave height and long duration of the 2011 tsunami in Sendai Bay^10^. The steep shoreface along Sendai Bay also reflected the tsunami^4,9^. These multiple reflections led to the long-term effect and high total energy. Although no information on the age of the upper sandy sediments were available, the huge energy of the 2011 tsunami probably reworked the sandy CGS sediments. High storm waves and human (fishery) activities in Sendai Bay are other candidates for reworking the CGS sediments. However, using the observational wave data (Supplementary Tables S6 and S7) and the sediment grain size of the CGS, the critical water depth for sand movement was calculated as < 50 m (Supplementary Table S8). This is shallower than the water depths (~ 100 m maximum) of the upper sand layer in our cores. The structure of the upper sand layers was similar (massive and without burrows) in all the obtained cores, suggesting that a similar process formed the structure. We believe that it is difficult to form such a similar structure by human (fishery) activities, such as bottom trawling, throughout the CGS area. Therefore, the seafloor disturbance by the tsunami wave with high frictional velocity could resuspend and transport large amounts of sand and mud on the inner shelf of Sendai Bay up to ~ 100 m water depth.

Several coarse-grained (sandy) layers were intercalated in the NISM and NWISM sediments and were interpreted as storm sand layers^24^. Although our redeposited layers lacked a sequence of basal coarse-grained part, the sediment characteristics of the upper portion were similar to those of reported storm sand layers^24^. A coarse-grained layer with similar sedimentary structures was formed in the muddy Onagawa Bay sediments, near Sendai Bay by the 2011 tsunami^43^. Previous studies also suggested the repeated occurrence of such coarse-grained layers^24,43^. Although the calculated recurrence intervals of the thick coarse-grained layers on the Sendai shelf (~ 100 years^24^) were slightly shorter than the intervals estimated from the onshore tsunami deposits on Sendai Plain^44,45^ and those from the deep-sea turbidites in the Japan Trench floor^46^, at least some coarse-grained layers may have formed by sediment remobilization similar to that of the 2011 Tohoku-oki tsunami. Interestingly, a muddy sediment with multiple coarse-grained layers and without bioturbation was formed by a single tsunami event at the northernmost Sendai Bay near the Ishinomaki coast at Site 2 (Fig. 3A). The complex behavior of the tsunami wave near the coastline might contribute to the formation of multiple coarse-grained layers. Moreover, the high sedimentation rate of the inner shelf mud (160–490 cm ky^−1^)^24^ might also play an important role in the effective preservation of such layers. Another evidence of the past sediment remobilizations was the intercalated thin mud layer in the coarse sand at Site 21 (Fig. 4B), which had similar characteristics with the surficial mud layer newly deposited by the 2011 tsunami. This also suggested that the coarse sand in CGS should have moved and covered the event-related mud layers. However, although a large storm wave is a candidate for sand remobilization, further consideration is needed.

Tsunami-induced shelf-mud resuspension is also important for offshore sedimentation because part of the resuspended mud is moved offshore to the slope as turbidity currents^17,18,21,22^. Although the 2011 event deposits on the lower slope and in the Japan Trench were diatomaceous and had no shallow marine origin signature^22,46^, the 869 Jogan Earthquake event deposits in the Japan Trench contained calcareous nannofossils^46^. This indicates that the origin of the turbidity currents that formed the 869 Jogan event deposits in the Japan Trench was the shelf–slope area above the calcium carbonate compensation depth (~ 4000–4500 m^47^). Our findings suggest that the formation of a larger turbid cloud in the shallow marine area by the 869 Jogan tsunami than the 2011 tsunami may have contributed to the larger volume and greater transport distance of the sediment grains of shallow marine origin to the deep sea.

The increase in the mud content near the mouths of the Abukuma and Natori rivers could be explained in two ways. The first is the muddy sediment input from the river mouth areas by the 2011 tsunami, where large coastal erosions occurred in 2011. However, the coastal sediments in this area^24,25^ and the reported 2011 tsunami deposits formed near the shoreface^19,20^ were sandy. Another possible origin of these muds could be a flood event between the 2011 tsunami and the survey period. Flood-induced fine sediment deposition is a possible mechanism that could change the surface sediment grain size near the river mouth^48,49^. In September 2011, Typhoon T1115 ROKE brought heavy rainfall to the upper reaches of the Abukuma and Natori rivers. The swollen rivers carried suspended terrigenous materials to the ocean. Such flood-related fine-grained sediments might thus deposit near the river mouths. The higher proportion of terrigenous organic carbon observed in the stable carbon isotope and C/N ratio of the post-2011 tsunami surface sediments in these areas^40,41^ could support this possibility. Moreover, the higher radiocesium activities (> 20 times larger than those at Sites 20 and 21) might be due to the adsorption of radiocesium on mica-like clay minerals and/or organic matters of terrigenous origin delivered to the ocean by this flood^50^.

This is the first study to examine the exact effect of an extreme wave event on the shelf sediments by comparing the pre- and post-event surface sediment data of a wide area. This study indicated the large impact of a huge tsunami, such as the Tohoku-oki tsunami in 2011, on the shallow water environments. The large friction velocity of the tsunami wave and the long-term effects of the tsunami might contribute to the large sediment reworking on the Sendai shelf. Such sediment reworking might have repeatedly occurred due to huge tsunamis. Such redeposited layers can also be preserved in the shelf mud sequence. The detection of ^134^Cs from the FDNPP in the redeposited mud indicated that the suspended shelf water condition was maintained for at least several days or more after the tsunami. Part of the resuspended mud moved offshore to the slope as turbidity currents^17,18,21,22^. Thus, a huge tsunami is an important mechanism not only for shelf sedimentation but also for deep-sea sedimentation along active plate margins.

## Methods

Fifty surface sediment samples (~ 1/3 of the sampling sites of the 1985 survey^24,25^) were collected from the inner to outer shelf of Sendai Bay (Fig. 1) from August 27 to September 8, 2012, i.e., ~ 1.5 years after the 2011 Tohoku-oki earthquake and tsunami, using a Smith-McIntyre grab sampler. The sampling locations and water depths are listed in Supplementary Table S1. We used the D-GPS system (Trimble SPS351) for ship positioning with an accuracy of ± 1 m to collect sediment samples from the same locations of the 1985 survey. However, the combination of radio navigation and radar positioning with an accuracy of a few tens of meters was used for the 1985 survey. Therefore, slight differences may be observed between the sampling positions of the two surveys.

Two square pillar plastic boxes were vertically pushed into the sediment from the surface to take the subcores. 5–16.5 cm-long subcores were obtained. The subcores were split along the length into working halves and 1-cm-thick slab samples were prepared for soft X-ray radiography at the Geological Survey of Japan, AIST. Cutting surface of the working half was visually described and photographed. Mud content was measured for the uppermost 0–1 cm (surface) sample taken on board from the obtained sediment surface by wet sieving using a 63 µm (4 ø) mesh. Residues (sand and gravels) were oven-dried, and were sieved at 0.5 ø intervals for 15 min. For the selected sites, subsamples for grain-size analysis were taken a 1-cm thick sample from the specific horizons. Grain-size analysis was done using the same method of the surface samples.

For the selected four sites, we sliced the working half core at 1-cm intervals for radioactivity measurements. The sliced samples were weighed and freeze-dried for more than 48 h. The water content (WC) was determined from the difference in the weight of the wet and freeze-dried sediment and expressed as the ratio (%) of the water weight to the dry sediment solid weight. The dry bulk density (DBD; g cm^−3^) of the sediment was calculated by assuming that the density of grains and interstitial water were 2.45 g cm^−3^ and 1.02 g cm^−3^, respectively, as

$$ {\text{DBD}} = {1}00/\left( {{1}00/{2}.{45} + {\text{WC}}\% /{1}.0{2}} \right). $$

The freeze-dried samples were stored in a plastic cylindrically shaped container with a 50 mm diameter. Radioactivities for ^134^Cs, ^137^Cs, ^210^Pb and ^214^Pb were measured using an ORTEC GEM-FX5825P4 low-background plane-shape Ge-semiconductor detector with a 58 mm diameter at Hokkaido University. The measurement time was around 15 h, and the gamma-ray spectra were obtained using a SEIKO EG&G MCA7600 multi-channel analyzer. The “excess ^210^Pb” is defined as the difference between the measured activities (Bq kg^−1^) of ^210^Pb (supported + unsupported) and ^214^Pb, which is equal to the activity of the supported ^210^Pb, assuming the uranium series has reached secular equilibrium^51^. The activity of each radionuclide was converted to the value as of September 1, 2012, assuming a half-life of 2.0 years for ^134^Cs, 30.1 years for ^137^Cs, and 22.3 years for ^210^Pb.

The critical water depth for sediment motion was determined by the following equation derived from flume experiments and natural observations^52^:

1 $$ {\text{H}}/{\text{H}}_{0} = \alpha \left( {{\text{d}}/{\text{L}}_{0} } \right)^{{\text{n}}} ({\text{sinh 2}}\pi {\text{h}}_{{\text{i}}} /{\text{L}})\left( {{\text{L}}_{0} /{\text{H}}_{0} } \right) $$

where d is the sediment grain size (m); H_0_ and L_0_ are the deep-water wave height and length (m), respectively; h_i_ is the wave base (m); α is 1.35 for group movement, in which most particles move in the same direction as a group, and 2.4 for complete movement, in which particle movement produces a change in water depth; and n is 1/3^52–54^. Based on observational data (Nationwide ocean wave information network for ports and harbours; https://nowphas.mlit.go.jp/pastdata/?lng=eng) of significant waves at Sendai New Port from 1981 to 2011 and off Sendai Bay (central Miyagi GPS ocean wave meter station; Latitude: 38°13.950′, Longitude: 141°41.017′, Water depth: 144 m) from 2009 to 2018 (Supplementary Tables S6 and S7), we used 6, 7, 8, and 9 m for H_0_, and 10, 12, and 15 s for the wave period (T). Using the following equation, we calculated the deep-water wavelength (L_0_) for each wave period.

2 $$ {\text{L}}_{0} = {\text{ gT}}^{{2}} /{2}\pi $$

The major grain size in the CGS was coarse to very coarse sand. Thus, we used 0.75, 1.0, 1.5, and 2.0 mm for the sediment grain size (d).

## Supplementary Information

Supplementary Information.
