# Supplementary material for: The 2011 Tohoku-oki tsunami-induced sediment remobilization on the Sendai shelf, Japan, from a comparison of pre- and post-tsunami surface sediments
Source: Sci Rep. 2021 Apr 12;11:7864. doi: 10.1038/s41598-021-87152-8 (PMC8041997; doi:10.1038/s41598-021-87152-8)
Supplement: Supplementary file 1 — Supplementary Information. [file 41598_2021_87152_MOESM1_ESM.pdf]

Supplementary information to:

The 2011 Tohoku-oki tsunami-induced sediment remobilization on the Sendai shelf, Japan, from a comparison of pre- and post-tsunami surface sediments

Ken Ikehara[1, *], Tomohisa Irino[2] and Yoshiki Saito[1, 3]

1 Geological Survey of Japan, National Institute of Advanced Industrial Science and Technology (AIST), Tsukuba Central 7, 1-1-1 Higashi, Tsukuba, 305-8567 Japan
2 Faculty of Environmental Earth Science, Hokkaido University, Kita 10, Nishi12, Kita-ku, Sapporo, 060-0810 Japan
3 Estuary Research Center, Shimane University, Nishikawatsu-cho 1060, Matsue, 690-8504 Japan

* Corresponding author: k-ikehara@aist.go.jp

Contents of this file

Supplementary Table S1
Supplementary Table S2
Supplementary Table S3
Supplementary Table S4
Supplementary Table S5
Supplementary Table S6
Supplementary Table S7
Supplementary Table S8
Supplementary Figure S1

Introduction

This supporting information provides additional tables and figure to describe the locations and water depths of sampling sites of the 2012 survey (Supplementary Table S1), and characteristics of pre- and post-tsunami surface sediment grain size (gravel, sand, and mud content; content of very coarse-, coarse-, medium-, fine-, and very fine-sand; mode of sand; mode change) (Supplementary Table S2), result of radioactivity

measurements for subcores at Sites 20, 21, 168, and 234 (Supplementary Table S3), list of large earthquakes, large precipitation and strong wind events at Sendai during 1985–2012 (Supplementary Table S4), list of major flooding events around Sendai Bay during 1985–2012 (Supplementary Table S5), list of high waves at Sendai New Port during 1981–2010 (Supplementary Table S6), list of high waves at GPS 0cean wave meter station of central Miyagi (Latitude: 38°13.950’, Longitude: 141°41.017’, water depth: 144 m) during 2009–2018 (Supplementary Table S7), result of calculation of the critical water depths for sand movements (Supplementary Table S8) and comparison of sedimentary structures of the 1985 and 2012 subcores (Supplementary Figure S1).

Supplementary Table S1 Sampling locations and water depths of the 2012 survey

Site	Latitude	Longitude	Water Depth (m)
2	38° 21.697′	141° 17.263′	20.55
4	38° 20.521′	141° 13.201′	19.71
6	38° 20.009′	141° 17.401′	27.13
8	38° 19.911′	141° 22.200′	29.34
12	38° 16.795′	141° 14.495′	33.22
13	38° 16.801′	141° 18.321′	39.73
14	38° 17.009′	141° 22.764′	42.45
19	38° 13.949′	141° 14.748′	41.95
20	38° 14.227′	141° 18.217′	41.37
21	38° 14.090′	141° 23.772′	51.23
22	38° 13.362′	141° 02.588′	22.34
24	38° 12.817′	141° 09.026′	32.11
28	38° 12.529′	141° 25.427′	65.22
37	38° 11.855′	141° 04.420′	26.79
39	38° 11.386′	141° 11.227′	37.35
41	38° 11.319′	141° 19.340′	45.60
43	38° 10.875′	141° 27.145′	82.40
52	38° 10.233′	141° 05.881′	30.92
54	38° 09.884′	141° 13.041′	39.55
56	38° 09.663′	141° 21.672′	56.28
58	38° 09.071′	141° 29.130′	103.13
67	38° 08.790′	141° 07.561′	34.00
69	38° 08.111′	141° 15.181′	42.20
80	38° 07.582′	141° 02.788′	29.03
82	38° 07.230′	141° 09.435′	35.67
84	38° 06.518′	141° 17.144′	47.60
86	38° 06.184′	141° 26.074′	99.04
94	38° 06.681′	140° 58.039′	22.50
96	38° 06.115′	141° 04.181′	30.86
98	38° 05.594′	141° 11.169′	38.68
108	38° 05.200′	141° 19.497′	57.04
110	38° 04.667′	141° 05.811′	33.78
112	38° 03.828′	141° 13.025′	41.22
120	38° 03.200′	141° 22.751′	82.34
122	38° 03.593′	141° 00.661′	28.59
124	38° 03.015′	141° 07.313′	36.65
134	38° 02.592′	141° 15.110′	45.11
136	38° 02.135′	141° 02.110′	31.27
147	38° 01.389′	141° 09.031′	38.70
149	38° 00.559′	141° 03.899′	33.04
159	38° 00.271′	141° 10.696′	39.68
161	37° 59.575′	140° 57.156′	22.90
168	37° 58.968′	141° 04.921′	33.75
170	37° 58.127′	141° 14.005′	47.45
172	37° 57.611′	140° 59.718′	26.27
181	37° 56.682′	141° 06.267′	32.36
188	37° 55.346′	141° 16.107′	52.27
190	37° 55.032′	141° 07.632′	33.44
224	37° 55.050′	140° 59.235′	19.22
234	37° 53.874′	141° 05.683′	30.85

Supplementary Table S2 Result of grain size analysis

Site	Year	Gavel content (wt.%)	Sand content (wt.%)	Mud content (wt.%)	very coarse sand (wt.%)	coarse sand (wt.%)	medium sand (wt.%)	fine sand (wt.%)	very fine sand (wt.%)	Mode of sand	Mode change
2	2012	0.00	10.64	89.36	0.02	0.24	0.44	1.12	8.81	very fine sand	no change
	1985	0.00	5.20	94.80	0.00	0.23	0.20	0.51	4.26	very fine sand	
4	2012	0.00	0.99	99.01	0.00	0.00	0.18	0.09	0.72	very fine sand	no change
	1985	0.00	17.04	82.96	0.00	0.23	0.65	1.60	14.56	very fine sand	
6	2012	0.00	0.61	99.39	0.00	0.00	0.14	0.11	0.36	very fine sand	no change
	1985	0.00	1.78	98.22	0.00	0.12	0.20	0.29	1.17	very fine sand	
8	2012	0.00	0.82	99.18	0.00	0.00	0.07	0.00	0.75	very fine sand	no change
	1985	0.00	0.72	99.28	0.00	0.00	0.12	0.20	0.40	very fine sand	
12	2012	0.00	1.43	98.57	0.00	0.00	0.19	0.00	1.24	very fine sand	no change
	1985	0.00	7.56	92.44	0.00	0.09	0.21	0.43	6.83	very fine sand	
13	2012	0.00	0.25	99.75	0.00	0.00	0.00	0.12	0.12	very fine sand	fining
	1985	0.00	38.90	61.10	0.00	2.78	16.97	13.26	5.89	medium sand	
14	2012	0.00	0.20	99.80	0.00	0.00	0.00	0.20	0.00	fine sand	coarsening
	1985	0.00	1.06	98.94	0.00	0.21	0.11	0.15	0.59	very fine sand	

19	2012	0.00	1.15	98.85	0.00	0.00	0.17	0.17	0.80	very fine sand	no change
	1985	0.00	1.94	98.06	0.00	0.06	0.09	0.16	1.63	very fine sand	
20	2012	0.10	37.64	62.26	0.46	1.31	17.00	12.85	6.02	medium sand	fining
	1985	42.91	56.62	0.47	22.89	19.94	11.23	2.11	0.46	very coarse sand	
21	2012	0.00	1.65	98.35	0.37	0.25	0.37	0.31	0.34	medium sand	fining
	1985	26.16	73.80	0.04	4.67	47.86	18.49	2.24	0.54	coarse sand	
22	2012	0.00	0.54	99.46	0.00	0.00	0.00	0.18	0.36	very fine sand	no change
	1985	0.00	3.29	96.71	0.00	0.11	0.30	0.47	2.52	very fine sand	
24	2012	0.00	18.03	81.97	0.13	1.41	2.58	5.08	8.82	very fine sand	fining
	1985	0.00	58.13	41.87	1.23	9.01	19.57	20.59	7.73	fine sand	
28	2012	0.00	18.54	81.46	0.03	0.63	2.84	10.01	5.02	fine sand	coarsening
	1985	0.00	4.04	95.96	0.00	0.13	0.20	1.61	2.10	very fine sand	
37	2012	0.00	2.46	97.54	0.00	0.07	0.33	0.60	1.46	very fine sand	no change
	1985	0.00	19.36	80.64	0.00	0.33	2.67	3.15	13.21	very fine sand	
39	2012	0.00	15.20	84.80	0.00	0.06	0.24	0.91	13.99	very fine sand	no change
	1985	0.00	19.31	80.69	0.00	0.20	1.05	1.53	16.53	very fine sand	
41	2012	6.79	90.97	2.24	15.88	43.88	29.05	2.09	0.07	coarse	no change

										sand	
	1985	13.10	86.81	0.09	27.36	40.27	17.09	1.94	0.15	coarse sand	
43	2012	0.00	4.85	95.15	0.00	0.00	0.17	2.72	1.95	fine sand	coarsening
	1985	0.00	2.85	97.15	0.00	0.06	0.10	0.79	1.90	very fine sand	
52	2012	0.46	97.83	1.71	0.95	17.44	64.03	14.53	0.87	medium sand	no change
	1985	0.00	46.30	53.70	0.77	6.21	19.11	10.69	9.52	medium sand	
54	2012	0.00	98.77	1.23	0.95	31.77	60.74	5.18	0.13	medium sand	coarsening
	1985	0.00	22.04	77.96	0.00	1.19	4.84	3.68	12.33	very fine sand	
56	2012	35.58	61.02	3.40	28.42	21.12	9.69	1.65	0.13	very coarse sand	no change
	1985	7.21	90.72	2.07	34.93	33.86	15.42	4.32	2.18	very coarse sand	
58	2012	0.00	19.69	80.31	0.13	0.26	0.85	3.47	14.98	very fine sand	fining
	1985	0.49	56.78	42.73	1.21	4.89	29.89	11.52	8.78	medium sand	
67	2012	0.00	84.99	15.01	0.00	4.10	45.20	30.00	5.70	medium sand	no change
	1985	0.00	61.95	38.05	0.00	2.73	22.97	14.47	21.78	medium sand	
69	2012	0.09	97.60	2.32	3.91	43.26	44.09	6.16	0.18	medium sand	no change
	1985	0.01	99.96	0.03	2.53	42.43	49.12	5.80	0.09	medium sand	

80	2012	0.02	71.96	28.02	0.21	0.85	7.13	22.56	41.21	very fine sand	no change
	1985	0.00	64.12	35.88	0.00	0.63	11.43	16.74	35.32	very fine sand	
82	2012	0.07	97.82	2.12	0.51	21.63	63.97	11.18	0.53	medium sand	no change
	1985	0.00	83.82	16.18	0.23	4.59	37.98	24.95	16.07	medium sand	
84	2012	2.19	97.07	0.74	38.79	49.53	6.68	1.94	0.13	very coarse sand	coarsening
	1985	7.92	92.04	0.04	19.14	50.56	20.31	1.94	0.09	coarse sand	
86	2012	0.00	43.87	56.13	0.27	2.61	21.17	12.21	7.61	medium sand	no change
	1985	0.20	31.59	68.21	0.36	1.86	12.95	7.43	8.79	medium sand	
94	2012	0.14	77.28	22.58	0.51	2.87	34.21	36.52	3.17	fine sand	fining
	1985	0.30	99.66	0.04	8.31	61.87	27.01	2.37	0.10	coarse sand	
96	2012	0.75	98.33	0.91	6.81	53.93	34.78	2.75	0.07	coarse sand	no change
	1985	0.93	99.04	0.03	6.27	50.26	36.15	6.12	0.24	coarse sand	
98	2012	0.00	97.00	3.00	0.62	7.99	39.69	44.55	4.15	fine sand	fining
	1985	0.65	99.18	0.17	3.53	34.43	45.11	15.02	1.09	medium sand	
108	2012	5.55	92.93	1.52	14.06	52.12	20.08	6.46	0.22	coarse sand	coarsening
	1985	0.64	91.05	8.31	1.20	4.45	36.68	42.26	6.46	fine sand	
110	2012	0.40	98.73	0.87	19.17	47.65	28.32	3.45	0.14	coarse sand	no change

	1985	6.92	93.04	0.04	28.16	36.59	22.98	5.16	0.15	coarse sand	
112	2012	1.28	88.07	10.65	1.84	16.22	46.32	20.08	3.62	medium sand	no change
	1985	0.45	89.48	10.07	2.20	20.51	44.62	18.36	3.79	medium sand	
120	2012	0.00	97.07	2.93	0.11	4.61	69.01	21.22	2.12	medium sand	fining
	1985	1.37	98.57	0.06	5.51	64.82	24.17	3.76	0.31	coarse sand	
122	2012	0.98	93.70	5.31	2.13	7.01	64.36	19.31	0.90	medium sand	fining
	1985	55.42	44.57	0.01	29.03	12.12	2.96	0.39	0.07	very coarse sand	
124	2012	0.27	98.74	0.99	17.29	50.56	28.58	2.23	0.08	coarse sand	no change
	1985	1.71	98.27	0.02	19.42	43.11	32.88	2.75	0.11	coarse sand	
134	2012	0.15	98.93	0.92	21.42	35.05	38.90	3.49	0.07	medium sand	fining
	1985	0.20	99.79	0.01	8.10	56.26	32.42	2.94	0.06	coarse sand	
136	2012	0.68	98.46	0.86	14.33	51.63	30.79	1.67	0.04	coarse sand	coarsening
	1985	0.67	99.15	0.18	1.34	20.37	67.57	9.56	0.31	medium sand	
147	2012	3.71	95.49	0.79	41.78	28.93	19.41	5.21	0.17	very coarse sand	coarsening
	1985	6.87	93.08	0.05	24.02	34.27	29.16	5.40	0.23	coarse sand	

149	2012	11.08	87.75	1.17	14.02	35.86	33.59	4.19	0.09	coarse sand	coarsening
	1985	0.04	99.92	0.04	1.66	36.76	53.94	7.32	0.24	medium sand	
159	2012	0.90	98.35	0.75	20.04	42.31	32.02	3.92	0.06	coarse sand	no change
	1985	0.24	99.71	0.05	18.55	50.88	24.98	5.06	0.24	coarse sand	
161	2012	57.20	42.11	0.69	25.88	11.18	4.43	0.56	0.06	very coarse sand	no change
	1985	72.12	27.83	0.05	12.45	8.12	5.31	1.75	0.20	very coarse sand	
168	2012	1.10	42.27	56.63	1.09	5.93	17.66	16.04	1.55	medium sand	fining
	1985	0.79	99.16	0.05	14.12	60.52	22.33	2.05	0.14	coarse sand	
170	2012	29.89	69.22	0.89	37.57	24.40	6.67	0.56	0.01	very coarse sand	no change
	1985	25.96	73.98	0.06	30.26	28.04	13.56	2.01	0.12	very coarse sand	
172	2012	0.00	98.75	1.25	2.68	43.64	47.20	5.08	0.14	coarse sand	coarsening
	1985	0.00	98.15	1.85	0.24	1.88	10.67	72.76	12.60	fine sand	
181	2012	29.44	69.76	0.80	27.20	28.83	12.83	0.85	0.04	coarse sand	fining
	1985	49.81	50.16	0.03	22.98	17.01	8.54	1.54	0.10	very coarse sand	

188	2012	1.24	97.89	0.87	21.97	47.07	27.10	1.72	0.04	coarse sand	
	1985									no data	
190	2012	6.35	92.52	1.13	18.20	41.09	29.81	3.36	0.06	coarse sand	no change
	1985	21.16	78.78	0.06	16.32	29.30	27.52	5.36	0.28	coarse sand	
224	2012	0.88	43.79	55.33	0.00	0.18	1.41	7.50	34.70	very fine sand	fining
	1985	0.14	87.88	11.98	0.25	3.32	38.79	35.15	10.37	medium sand	
234	2012	0.00	16.71	83.29	0.00	0.00	0.91	6.53	9.27	very fine sand	fining
	1985	0.03	83.19	16.78	0.28	1.83	34.17	35.02	11.89	fine sand	

Supplementary Table S3 Result of radioactivity measurements

Site	Sampling Depth (cm)		Water content (%)	excess 210Pb (Bq/kg)	134Cs (Bq/kg)	137Cs (Bq/kg)	134Cs/137Cs at Sept. 1, 2012	134Cs/137Cs at Mar. 15, 2011
20	0	0.5	72.7	249.9	53.3	103.6	0.51	0.81
	0.5	1.0	74.8	94.4	21.0	41.1	0.51	0.81
	1	1.5	80.5	100.3	12.1	26.5	0.46	0.72
	1.5	2.0	80.0	85.6	9.3	19.1	0.49	0.77
	2	2.5	76.9	102.0	6.2	14.6	0.43	0.67
	2.5	3.0	75.6	113.8	7.2	13.9	0.52	0.83
	3	3.5	69.7	93.1	4.5	7.5	0.59	0.94
	3.5	4.0	40.5	58.5	6.6	12.8	0.52	0.82
	4	4.5	40.0	70.1	0.0	4.5	0.00	
	4.5	5.0	27.0	45.8	0.0	3.8	0.00	
21	0	0.5	134.1	127.9	66.0	114.9	0.57	0.91
	0.5	1.0	134.2	127.3	51.4	107.8	0.48	0.76
	1	1.5	144.1	124.1	89.1	161.7	0.55	0.87
	1.5	2.0	136.4	87.3	52.3	99.1	0.53	0.84
	2	2.5	115.0	93.2	31.5	61.1	0.52	0.82
	2.5	3.0	119.3	91.7	15.4	33.9	0.46	0.72
	3	3.5	91.9	78.6	9.1	15.5	0.58	0.92
	3.5	4.5	38.9	40.7	2.2	4.3	0.50	0.79
	4.5	5.5	17.6	21.0	0.0	0.0		
	5.5	6.3	17.9	29.9	0.0	0.0		
	6.25	6.75	59.8	132.3	0.0	0.0		
	6.75	7.75	22.4	34.5	0.0	0.0		
	7.75	8.75	14.5	5.2	0.0	0.0		
168	0	0.5	164.5	225.4	1373.4	2661.3	0.52	0.82
	0.5	1.0	179.4	247.5	1497.9	2858.3	0.52	0.83
	1	1.5	169.7	150.2	1511.9	2899.6	0.52	0.83
	1.5	2.0	185.6	176.3	2203.3	4141.4	0.53	0.84
	2	2.5	209.6	235.4	2372.6	4525.6	0.52	0.83
	2.5	3.0	186.2	214.9	2699.5	5094.8	0.53	0.84
	3	4.0	226.8	208.6	3405.3	6427.2	0.53	0.84
	4	5.0	246.0	218.9	3719.5	7018.0	0.53	0.84
	5	6.0	219.8	205.1	3902.4	7342.4	0.53	0.84
	6	7.0	140.8	180.2	2626.9	4923.0	0.53	0.85
	7	8.0	52.5	58.8	1051.6	1964.5	0.54	0.85
	8	9	36.6	25.5	305.1	558.2	0.55	0.87
	9	10	44.9	7.4	167.9	305.1	0.55	0.87
	10	11	58.4	11.3	93.0	171.6	0.54	0.86
234	0	0.5	115.3	157.0	967.1	1815.7	0.53	0.84
	0.5	1.0	100.5	107.6	812.2	1534.0	0.53	0.84
	1	1.5	105.2	135.5	809.5	1550.0	0.52	0.83
	1.5	2.0	82.9	83.2	544.9	1011.7	0.54	0.85
	2	2.5	100.5	97.9	424.3	784.4	0.54	0.86
	2.5	3.0	118.6	114.6	421.5	790.1	0.53	0.84
	3	4.0	118.3	135.3	237.3	449.1	0.53	0.84
	4	5.0	97.8	119.2	95.1	175.5	0.54	0.86
	5	5.5	46.5	42.1	34.0	62.2	0.55	0.87
	5.5	6.5	130.4	71.4	23.7	47.3	0.50	0.79
	6.5	7.5	108.6	72.3	10.8	19.2	0.56	0.89
	7.5	8.5	65.5	29.6	2.9	6.9	0.42	0.67
	8.5	9.5	124.3	32.0	5.0	8.0	0.62	0.98

Supplementary Table S4 List of large earthquakes, large precipitation and strong wind events at Sendai during 1985–2012

Earthquake (Seismic Intensity at Sendai >5-)

Date	Seismic intensity at Sendai	Location of epicenter	Mj
2003/5/26	5-	off Miyagi	7.1
2003/7/26	5-	central Miyagi	6.4
2005/8/16	5+	off Miyagi	7.2
2008/6/14	5+	inland south Iwate	7.2
2011/3/11	6+	Tohoku-oki	9.0
2011/4/7	6+	off Miyagi	7.2
2012/8/30	5+	off Miyagi	5.6

Daily Precipitation at Sendai (>120.0 mm)

Date	Precipitation (mm)	Corresponding Typhoon
1986/8/5	296.0	T8610 SARAH
1990/9/20	154.0	T9019 BECKY
1994/9/22	147.0	
2000/7/8	124.0	T0003 KIROGI
2010/12/22	128.5	
2011/5/30	121.0	
2011/9/21	235.0	T1115 ROKE
2012/5/3	130.5	

Maximum Wind Speed at Sendai (>20.0 m/s)

Date	Maximum Wind speed (m/s)	Wind direction	Corresponding Typhoon
1987/3/25	21.6	W	
1994/9/30	21.1	SE	T9426 ORCHID
1995/11/8	20.6	WNW	
1997/2/26	20.6	WNW	
1997/3/11	24.0	WNW	
1998/9/16	20.0	SSE	T9805 STELLA
2012/4/4	20.7	W	

Supplementary Table S5 List of major flooding events around Sendai Bay during 1985–2012

Abukuma River (Station: Tateyama) Duration: 1998–2012

Date	Maximum Water Level (m)	Corresponding Typhoon or Precipitation Event
2002/7/11	24.18	T0205 RAMMASUN, T0206 CHATAAN & Baiu
2011/9/22	23.17	T1115 ROKE
2007/7/15	21.84	T0704 MAN-YI & Baiu
2006/10/7	21.76	
1999/9/16	21.76	T9916 ZIA & T9918 BART
2004/10/21	21.75	T0423 TOKAGE
1998/9/16	21.48	T9805 STELLA
1999/7/14	21.18	Baiu
2004/10/10	21.15	T0422 MA-ON
1999/6/30	20.94	Baiu

Abukuma River (Station: Fuseguro) Duration: 1985–2012

Date	Maximum Water Level (m)	Corresponding Typhoon or Precipitation Event
2002/7/11	5.53	T0205 RAMMASUN, T0206 CHATAAN & Baiu
1986/8/5	5.47	T8610 SARAH
1998/8/30	5.08	(T9804 REX)
2011/9/22	4.81	T1115 ROKE
1991/9/19	3.66	T9117 KINNA, T9118 LUKE, T9119 MIREILLE
1989/8/6	3.64	T8913 MAC
1998/9/16	3.38	T9805 STELLA
1994/9/30	3.33	T9426 ORCHID
1999/9/15	3.32	T9916 ZIA & T9918 BART
2007/7/15	3.28	T0704 MAN-YI & Baiu

Natori River (Station: Yuriage No. 2) Duration: 1999–2012

Date	Maximum Water Level (m)	Corresponding Typhoon or Precipitation Event
2002/7/11	2.93	T0206 CHATAAN & Baiu
2011/9/21	2.46	T1115 ROKE
2010/12/22	2.41	
2012/6/20	2.33	T1204 GUCHOL & Baiu
2012/5/3	2.30	
2006/10/7	2.27	
2006/12/27	2.24	
2007/7/15	2.17	T0704 MAN-YI & Baiu
2009/10/8	1.77	T0918 MELOR
1999/8/15	1.72	Tropical Low

Naruse River (Station: Nobiru) Duration: 1985–2012

Date	Maximum Water Level (m)	Corresponding Typhoon or Precipitation Event
2002/7/11	4.48	T0206 CHATAAN & Baiu
1994/9/30	4.34	T9426 ORCHID
1997/6/29	4.10	T9707 OPAL & Baiu
1998/9/16	4.08	T9805 STELLA
1989/8/28	4.07	T8917 ROGER
1990/12/1	4.02	
1990/9/20	4.00	T9019 FLO
1989/8/7	3.99	T8913 MAC
1998/8/30	3.93	(T9804 REX)
2006/10/7	3.86	

Supplementary Table S6 List of high waves at Sendai New Port during 1981–2011

Year	Date	Hour	Significant Wave Height (m)	Significant Wave Period (sec)	Corresponding Typhoon
2002	2-Oct	2	5.63	11.4	T0221 HIGOS
2007	7-Sep	12	5.55	10.5	T0709 FITOW
1982	20-Oct	10	5.36	9.9	
1982	20-Oct	12	5.32	10.6	
2002	2-Oct	0	5.28	9.1	T0221 HIGOS
2006	7-Oct	0	5.00	12.2	
1983	18-Feb	18	4.99	13.3	
1999	28-Oct	0	4.93	6.3	
2007	7-Sep	14	4.93	10.0	T0709 FITOW
2006	27-Dec	8	4.91	10.3	
1999	28-Oct	4	4.89	10.7	
2006	7-Oct	4	4.89	13.5	
2010	22-Dec	14	4.88	9.3	
1982	20-Oct	14	4.87	11.0	
1989	6-Aug	22	4.86	10.9	T8913 MAC
1990	4-Nov	20	4.74	9.2	
2003	8-Mar	2	4.72	11.6	
1993	14-Nov	12	4.71	10.5	
1982	20-Oct	8	4.61	9.1	
2002	21-Jan	20	4.60	9.4	
2010	22-Dec	16	4.59	9.9	
2006	6-Oct	22	4.58	11.8	
2006	7-Oct	2	4.57	12.6	
2002	21-Jan	22	4.54	10.3	
2002	22-Jan	0	4.53	10.9	
1983	9-Feb	0	4.51	13.5	

Supplementary Table S7 List of high waves at GPS 0cean wave meter station of central Miyagi (Latitude: 38°13.950’, Longitude: 141°41.017’, water depth: 144 m) during 2009–2018

Year	Date	Hour	Significant Wave Height (m)	Significant Wave Period (sec)	Corresponding Typhoon
2016	30-Aug	14	9.18	12.2	T1610 LIONROCK
2016	18-Jan	22	8.41	11.8	
2013	16-Oct	10	8.36	9.7	T1326 WIPHA
2013	16-Oct	14	8.33	13.0	T1326 WIPHA
2011	1-Jan	12	8.31	13.2	
2009	31-Jan	18	8.14	10.9	
2016	18-Jan	20	8.12	11.4	
2013	16-Oct	16	8.06	14.1	T1326 WIPHA
2014	15-Feb	20	8.04	11.2	
2011	1-Jan	8	7.97	12.6	
2016	30-Aug	16	7.85	11.9	T1610 LIONROCK
2016	30-Aug	12	7.75	11.7	T1610 LIONROCK
2014	15-Feb	22	7.74	11.2	
2014	15-Feb	18	7.68	10.7	
2014	9-Feb	12	7.52	11.2	
2009	31-Jan	16	7.44	10.4	
2009	1-Feb	2	7.44	12.3	
2009	31-Jan	20	7.43	11.0	
2014	9-Feb	10	7.43	11.8	
2014	15-Feb	16	7.40	10.2	

Supplementary Table S8 Result of calculation of the critical water depths for sand movements

Wave height (m)	Wave period (sec)	Sediment grain size (mm)	Wave base: General movement (m)	Wave base: Complete movement (m)	Wave height (m)	Wave period (sec)	Sediment grain size (mm)	Wave base: General movement (m)	Wave base: Complete movement (m)
6	10	0.75	25.3	13.4	8	10	0.75	33.0	18.7
6	12	0.75	28.4	14.4	8	12	0.75	38.2	20.4
6	15	0.75	31.5	15.4	8	15	0.75	43.8	22.2
6	10	1.00	23.1	11.9	8	10	1.00	30.4	16.8
6	12	1.00	25.5	12.7	8	12	1.00	34.7	18.2
6	15	1.00	28.0	13.7	8	15	1.00	39.3	19.6
6	10	1.50	19.9	10.1	8	10	1.50	26.8	14.4
6	12	1.50	21.8	10.7	8	12	1.50	30.1	15.4
6	15	1.50	23.9	11.4	8	15	1.50	33.7	16.6
6	10	2.00	17.9	9.0	8	10	2.00	24.4	12.8
6	12	2.00	19.4	9.5	8	12	2.00	27.1	13.7
6	15	2.00	21.0	10.1	8	15	2.00	30.0	14.6
7	10	0.75	29.4	16.3	9	10	0.75	36.3	21.3
7	12	0.75	33.4	17.3	9	12	0.75	42.6	23.4
7	15	0.75	37.7	18.8	9	15	0.75	49.8	25.6
7	10	1.00	26.9	14.4	9	10	1.00	33.6	19.2
7	12	1.00	30.2	15.4	9	12	1.00	39.0	20.9
7	15	1.00	33.7	16.6	9	15	1.00	44.8	22.8
7	10	1.50	23.5	12.2	9	10	1.50	29.9	16.5
7	12	1.50	26.0	13.1	9	12	1.50	34.1	17.8
7	15	1.50	28.7	14.0	9	15	1.50	38.6	19.2
7	10	2.00	21.2	10.9	9	10	2.00	27.4	14.7
7	12	2.00	23.3	11.6	9	12	2.00	30.9	15.8
7	15	2.00	25.5	12.4	9	15	2.00	34.5	17.1

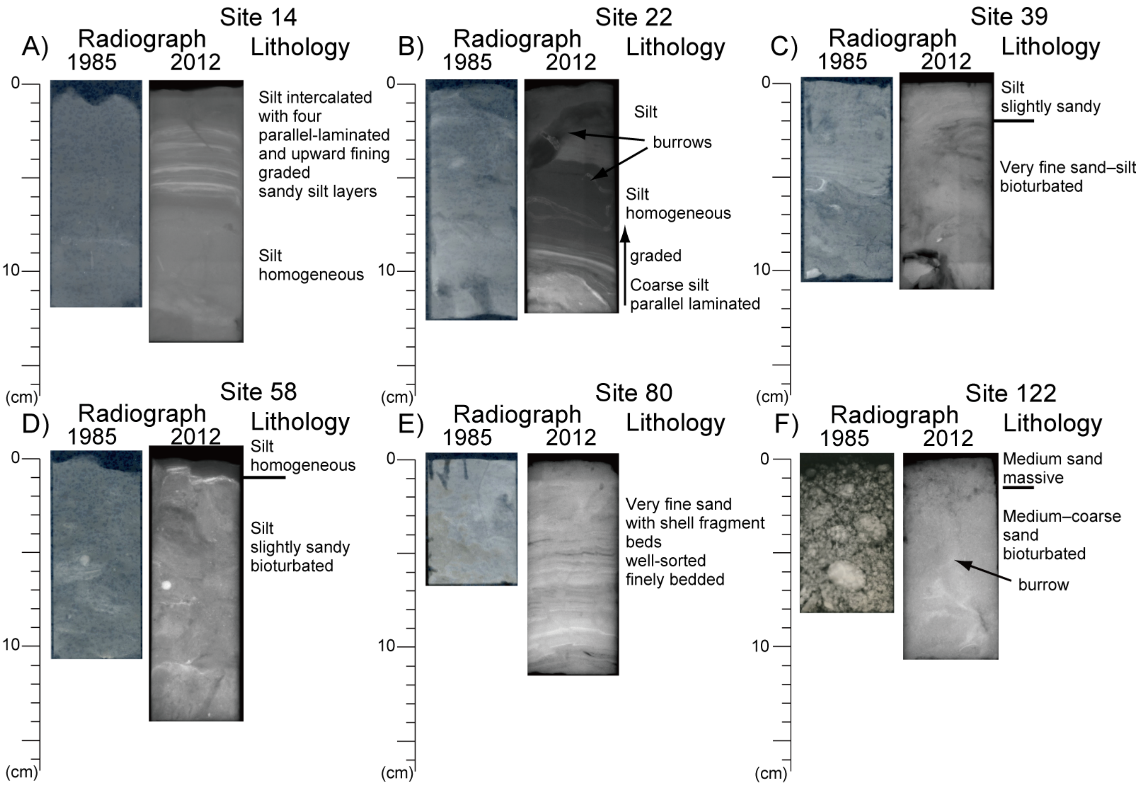

Supplementary Fig. S1 Comparison of sedimentary structures of the 1985 and 2012 sample. Site 14 (A) from the NISM, Sites 22 (B) and 39 (C) from the NWISM, Site 58 (D) from the MSM, and Sites 80 (E) and 120 (F) near the western margin of CGS.
